# Surface kinematic and depth-resolved analysis of human vocal folds *in vivo* during phonation using optical coherence tomography

**DOI:** 10.1117/1.JBO.26.8.086005

**Published:** 2021-08-19

**Authors:** Giriraj K. Sharma, Lily Y. Chen, Lidek Chou, Christopher Badger, Ellen Hong, Swathi Rangarajan, Theodore H. Chang, William B. Armstrong, Sunil P. Verma, Zhongping Chen, Ram Ramalingam, Brian J.-F. Wong

**Affiliations:** a University of California, Irvine Medical Center, Department of Otolaryngology–Head and Neck Surgery, Irvine, California, United States; b University of California, Irvine, Beckman Laser Institute and Medical Clinic, Irvine, California, United States; c OCT Medical Imaging, Inc., Irvine, California, United States; d University of California, Irvine, School of Medicine, Irvine, California, United States; e University of California, Irvine, Department of Biomedical Engineering, Irvine, California, United States

**Keywords:** optical coherence tomography, image processing, larynx, mucosal wave, tissues, *in vivo* imaging

## Abstract

**Significance:** The human vocal fold (VF) oscillates in multiple vectors and consists of distinct layers with varying viscoelastic properties that contribute to the mucosal wave. Office-based and operative laryngeal endoscopy are limited to diagnostic evaluation of the VF epithelial surface only and are restricted to axial-plane characterization of the horizontal mucosal wave. As such, understanding of the biomechanics of human VF motion remains limited.

**Aim:** Optical coherence tomography (OCT) is a micrometer-resolution, high-speed endoscopic imaging modality which acquires cross-sectional images of tissue. Our study aimed to leverage OCT technology and develop quantitative methods for analyzing the anatomy and kinematics of *in vivo* VF motion in the coronal plane.

**Approach:** A custom handheld laryngeal stage was used to capture OCT images with 800 A-lines at 250 Hz. Automated image postprocessing and analytical methods were developed.

**Results:** Novel kinematic analysis of *in vivo*, long-range OCT imaging of the vibrating VF in awake human subjects is reported. Cross-sectional, coronal-plane panoramic videos of the larynx during phonation are presented with three-dimensional videokymographic and space-time velocity analysis of VF motion.

**Conclusions:** Long-range OCT with automated computational methods allows for cross-sectional dynamic laryngeal imaging and has the potential to broaden our understanding of human VF biomechanics and sound production.

## 1 Introduction

The human vocal fold (VF) is a multilayered, three-dimensional (3D) structure that oscillates in multiple directions during phonation. In 1974, Hirano^1^ first described the cover-body theory of VF vibration as the complex interfacing of loose, superficial VF layers with more rigid, deeper strata. The varying viscoelastic properties of these layers play a fundamental role in producing the VF mucosal wave and modulating sound quality. Speech pitch is related to the frequency (Hz) of vibration which is, in turn, determined by the length, tension, and vibrational speed of the VF. Meanwhile, the loudness (dB) or intensity of voice is in part related to the amplitude of VF vibration. These human voice properties are largely determined by subepithelial components of the mucosal wave.

Indirect or direct imaging of the larynx *in vivo* by means of dental mirror laryngoscopy, flexible fiber-optic, distal chip, or rigid endoscopy is limited to characterization of the VF epithelial surface only. Current cross-sectional laryngeal imaging modalities including computed tomography, magnetic resonance, or B-mode ultrasound^2^^–^^4^ feature inadequate spatial and temporal resolution to characterize the VF substructure and mucosal wave propagation, respectively. Hence, our understanding of the biomechanics of subepithelial VF layers during phonation remains limited and is largely based on *ex vivo* and laryngeal model studies.^5^^–^^9^

In order to better understand and quantify the mechanics of VF motion, cross-sectional high-speed imaging of the VF remains a missing link. During phonation, as air flows cephalically out of the larynx, VF tissues successively lateralize and inferior tissue layers collapse medially, resulting in a phase difference which constitutes the vertical component of the mucosal wave. Contemporary *in vivo* functional laryngeal imaging modalities—including videostroboscopy, videokymography (VKG), and high-speed digital imaging (HSDI)—are restricted to axial-plane surface video of the superior mucosal wave in its anterior–posterior and transverse vectors.^10^^,^^11^ These technologies are often combined together or supplemented with indirect mucosal wave measurement techniques such as photoglottography, electroglottography (EGG), and color Doppler imaging to yield objective parameters including glottal area change, degree of contact between left vocal fold (LVF) and right vocal fold (RVF) and mucosal wave velocity, respectively.^11^^,^^12^ Although videostroboscopy with a flexible distal chip endoscope or rigid angled telescope may permit inferences about the caudal surfaces of the VF, *in vivo* coronal-plane imaging of the inferior, vertical component of the mucosal wave is not clinically feasible with any existing commercial clinical imaging technology.

Optical coherence tomography (OCT) is a high-resolution (10 to 15  μm), high-speed (50 kHz to 1.2 MHz) imaging modality which acquires cross-sectional images of biological tissue with up to 1.0 mm optical penetration depth.^13^^,^^14^ Early generations of laryngeal OCT systems were restricted to contact or near-contact mode imaging, requiring general anesthesia, and surgical endoscopy to image the human larynx in only a static state.^15^^,^^16^ As OCT systems evolved with long-range coherence length image capabilities (≥10  cm), office-based laryngeal imaging in awake patients was described. However, these early studies were limited by low imaging speeds (frame rates) or poor image quality.^17^^–^^20^ Recently, vertical cavity surface-emitting laser (VCSEL) technology, with output power between 10 and 20 mW, has permitted extended distance range (15 to 20 cm), panoramic imaging of the VF with improved resolution and faster imaging speeds.^14^^,^^21^ At a brief glance, existing usages of VCSEL OCT *in vivo* include visualizations of the human upper airway (nasopharynx, oropharynx, and supraglottis),^22^ microvascular angiography imaging of human oral cavity lesions,^23^ and volumetric OCT vibrometry measurements within mouse organ of Corti.^24^ In 2016, *in vivo* VCSEL OCT of human VF was first introduced: Coughlan et al.^25^ demonstrated its imaging capabilities with phonating VF, and Garcia et al.^26^ analyzed VF development and lesions^27^ in pediatric patients.

To build upon this technology, we evaluated VCSEL OCT of the phonating VF and now introduce quantitative methods for anatomical and kinematic analysis of the mucosal wave in the coronal plane of section. We then explore potential clinical applications of our imaging and analytical methods, which are pertinent to laryngologists and speech pathologists.

## 2 Methods

### 2.1 Participants

A prospective study was conducted to evaluate VCSEL OCT of the human VFs (n=33). Volunteer adult participants (age>18  years) with normal, healthy upper airways were recruited for participation. Exclusion criteria included participants with a history of laryngeal pathology or voice, speech, or swallowing complaints. Individuals were not excluded on the basis of race or gender. Potential participants were first screened for favorable upper airway anatomy by undergoing indirect mirror laryngeal examination. Individuals with a very low palatal arch, large oral tongue, posteriorly displaced base of tongue, or severe gag reflex—which would preclude office-based, transoral endoscopic imaging of the larynx—were excluded from participation. This study was approved by the human subjects Institutional Review Board at the University of California Irvine (HS #2003-3025). All participants provided written informed consent for participation.

### 2.2 OCT System Specifications

**Fig. 1:**
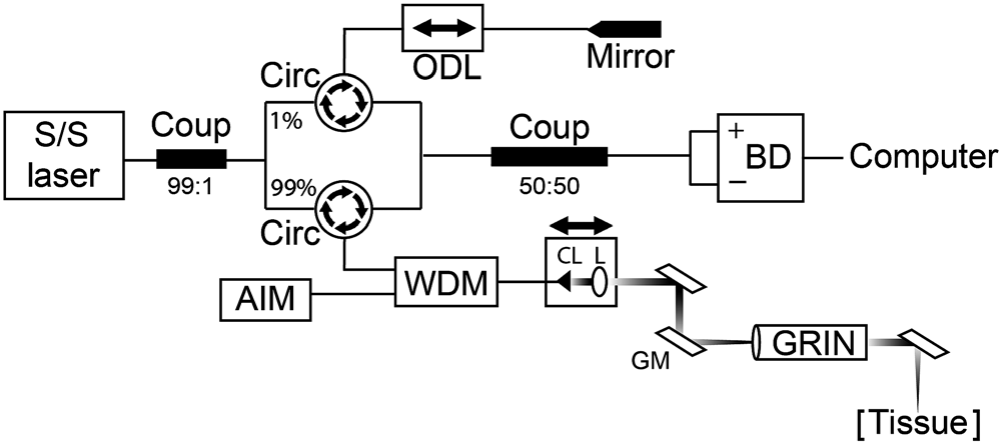
VCSEL OCT system schematics. S/S, swept source; Coup, coupler; Circ, fiber-optic circulator; ODL, optical delay line; BD, balanced detector; AIM, aiming laser; WDM, wavelength division multiplexer; CL, collimating lens; L, imaging lens; GM, Galvo mirrors; and GRIN, gradient index lens rod.

Specifications for the VCSEL OCT system have been previously reported, with only the pertinent details provided here.^25^ The OCT system consisted of a 1310-nm center wavelength 200 kHz VCSEL swept source (SL1310V1-20048, Thorlabs, Inc.; MA, USA) (Fig. 1). Output light was split by a 99:1 ratio fiber-optic coupler into sample and reference arms, respectively. In the reference arm, a fiber-optic pigtail faraday mirror was incorporated to reflect the reference beam. This served to minimize changes to the polarization state of light induced by external perturbations (e.g., vibrations) and, in turn, minimize intensity variations in the OCT images. The system and handheld laryngeal scanning stage were designed to allow the operator to manually adjust the sample arm length in real-time to focus the signal on the tissue of interest. To compensate for these changes in the sample arm path length, a motorized optical delay line (ODL) was used in the reference arm. Thus path length was adjusted by computer operator through the software, which in turn moved the hardware.^25^ In the sample arm, a visible 635-nm aiming laser beam was coupled into the OCT system using a wavelength division multiplexer (WDM) positioned between port 2 of the sample arm circulator and the laryngeal scanner setup. Combining the laser beams prior to the laryngeal scanner permitted the operator to visualize the OCT frames in real-time to identify the region of interest (ROI).

**Fig. 2:**
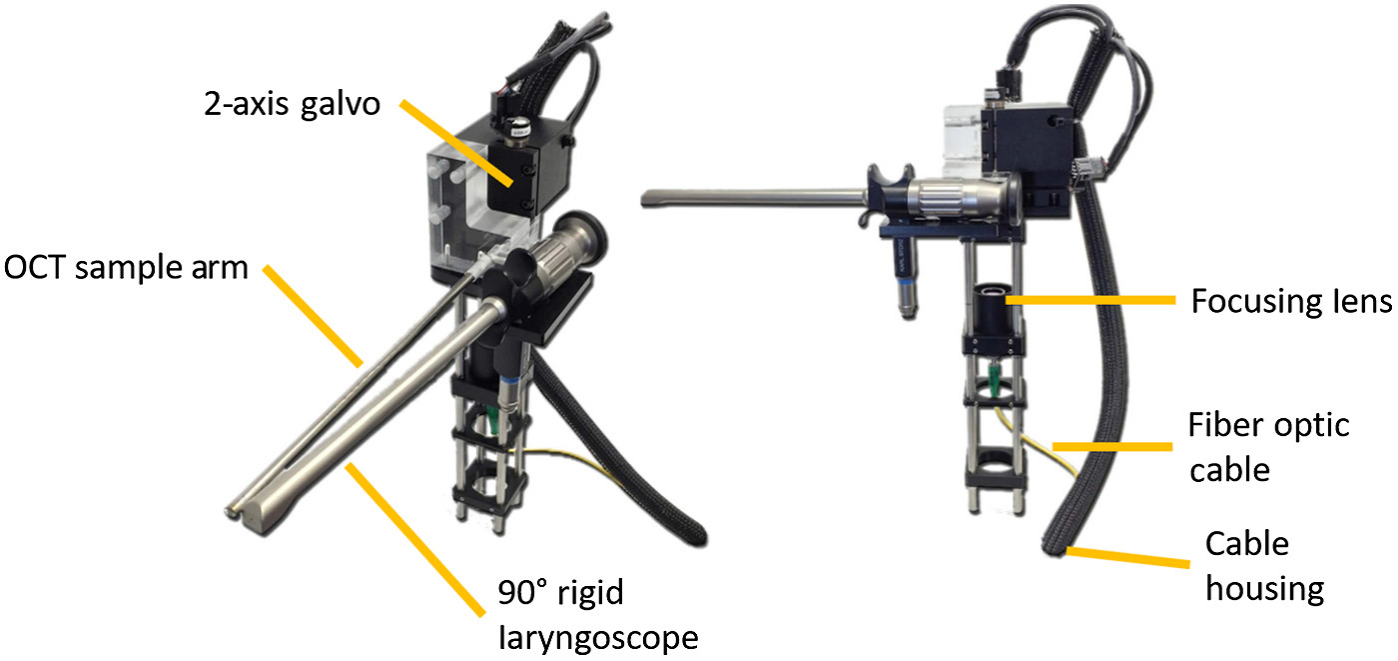
VCSEL OCT handheld laryngeal stage.^25^ The 1834-g VCSEL handpiece containing the rigid 90-deg endoscope and OCT sampling arm, within which housed a GRIN lens rod and 45-deg reflector. This figure was adapted from Ref. 25 and published under the CC BY 4.0 license (Video S1, 35.15 MB, MP4 [URL: https://doi.org/10.1117/1.JBO.26.8.086005.1]).

The handheld laryngeal stage (Fig. 2)^25^ consisted of a fiber collimator, a focusing lens, a two-axis galvo, a gradient index (GRIN) lens rod, and a 45-deg reflector to allow for transoral imaging of the VF from the oropharynx. To protect the optical components, a stainless-steel tube (5.2 mm outer diameter) was used to house the GRIN lens rod and the reflector. The aforementioned WDM output beam was transmitted through the collimator. The collimated output beam was focused by an achromatic doublet (f=125  mm), scanned and reflected off the two-axis galvo mirrors and subsequently directed down the one-pitch GRIN lens rod. At the end of the GRIN lens rod, a 45-deg reflector redirected the beam 90-deg toward the larynx. The power of incident light on the VFs was 10 mW.

With the fiber collimator and achromatic doublet mounted in the same subassembly, the operator was able to manually adjust the optical subassembly directly from the handheld stage to change the working distance of the sampling arm to match the position of the VF. The focal plane was preset to 60-mm distance from the 45-deg reflector, with an adjustable range of 30 to 100 mm to accommodate for intersubject variation in pharyngeal anatomy (distance between mid-oropharynx and VF). The beam spot size was 74.5  μm and corresponding depth of focus was 6.4 mm as measured by a beam profiler (DataRay Inc., Bella Vista, California). Based on the 12-mm OCT imaging range afforded by the use of the VCSEL swept source, this preset configuration allowed more than half of the vertical imaging range to be considered in focus. Using a single reflector as the test sample, the axial resolution of the OCT system was measured to be ∼12.5  μm in air, or ∼9.3  μm in tissue depending on the refractive index of tissue. Because the laryngeal scanner setup had a two-axis galvo positioned in between the focusing lens and the optical relay GRIN lens rod, the lateral scanning distance varied with the distance from the reflector, causing the cross-sectional scanning area to be fan shaped. This configuration, similar to a microelectromechanical system scanner in front of the GRIN lens rod, was chosen because a telecentric scanning setup would not fit within the dimensions of the human oropharynx. For the preset 60 mm working distance from the tip of laryngeal setup, the scanning range varied from 7.8 mm at the top to 9.2 mm at the bottom of an OCT image. For this study, a fixed lateral frame size of 800 A-lines was selected to achieve a frame rate of 250 Hz, the maximum frame rate of the system.

Transoral laryngeal OCT and video endoscopy were performed simultaneously. A conventional 90-deg rigid endoscope (15 cm length; Karl Storz SE & Co., Tuttlingen, Germany) was mounted adjacent to the GRIN lens rod in a handheld bracket (Fig. 2).^25^ This allowed the user to aim the OCT signal at an ROI that was approximately centered in the endoscopy video frame. The conventional endoscope does not emit infrared light (IR λ: 750 to 1400 nm) and thus did not interfere with OCT sampling.

### 2.3 Clinical OCT Imaging

An otolaryngologist-head and neck surgeon operated the handheld laryngeal OCT imaging stage and performed transoral laryngeal endoscopy while an engineer operated the software interface on a separate computer. All imaging was performed in an outpatient otolaryngology clinic. Assembly of the portable system took up to 15 min. Software launch and setups, including optical parameter adjustments, were typically completed within 2 min. Recording to acquire one data set could be completed within seconds, though longer imaging periods could be accomplished depending on the participants’ comfort level. In practice, a trained clinical user would be able to assist in the procedure as it follows the workflow of transoral video laryngoscopy. Video S1 highlights the VCSEL OCT system hardware and clinical imaging setup.

The video endoscopy tower and OCT computer were positioned behind the clinical examination chair, with both monitors in view of the surgeon. The oropharynx was topically anesthetized using 4% lidocaine delivered via atomizer. With the subject seated upright in the examination chair with the mouth open, the tongue was grasped and held outside the mouth by the surgeon using the non-dominant hand, as in conventional transoral laryngeal examination. The endoscope tip and GRIN rod mirror were treated with defogger solution and subsequently advanced in tandem through the oral airway using the dominant hand. Once the VFs were visualized within the endoscopy video frame, the surgeon manually adjusted the sampling arm (30 to 100 mm range) as necessary to center the focal plane at the VF along the y axis of the OCT frame. At this point, OCT image acquisition began. The patient was asked to breathe normally and then phonate the letter “e” at a normal speaking volume while taking breaths in between. During phonation, endoscopy video was simultaneously recorded. Multiple data sets were acquired per subject, as tolerated.

### 2.4 Image Postprocessing

All data sets were reviewed by an otolaryngologist-head and neck surgeon with experience in OCT image acquisition and analysis (G. K. S. and B. J. W.). Data sets selected for analysis featured OCT frames containing the entire width of bilateral VF, optimal signal-to-noise ratio, and soft tissue substructural resolution, high clarity of tissue contours and at least 3 s of consecutive OCT frames during phonation. All images acquired depicted the VF in the coronal anatomical plane, with the y axis of the frame representing craniocaudal height of the larynx. Using a stage calibration setup described later in this report (2.8. OCT measurement validation), axial pixel resolution was determined to be 5.94  μm/pixel in air. A refractive index of 1.4 was used to approximate the refractive index of human soft tissue,^29^^,^^30^ yielding an approximation of 4.24  μm/pixel axial resolution in tissue. Lateral pixel resolution is a function of the focusing optics of the OCT system and, in the present device with the reported settings used, the lateral pixel resolution was determined to be 10.625  μm/pixel. All images were postprocessed with a 2.50:1 width-to-height ratio to approximate the differences in axial and lateral resolutions in tissue.

Raw interferometer spectral data were processed using fast Fourier transforms based on Cooley–Tukey algorithm and Bluestein algorithm in the custom acquisition software (Full Range OCT; Irvine, CA) to yield grayscale images. Individual data sets (2000+ frames) were cropped to frames of interest which included the VF during normal respiration and at least 3 s (≥600  frames) of phonation. Given the vibration of the operator’s hand and patient movement, the vertical position of the VF within the coronal image frame would fluctuate slightly. To correct for this, image subsets were stabilized using an OpenCV template matching plug-in in ImageJ (National Institutes of Health; Bethesda, MD).^31^^,^^32^ Next, all frames within the subset were cropped to an ROI (VF and laryngeal ventricle only) to reduce memory requirements and processing load. Subsequent analysis was completed in Python (Python Software Foundation; Beaverton, OR), with each sessions’ cropped subset (up to 500 MB) analyzed in seconds to a couple minutes depending on the total number of frames. Cropped grayscale images were smoothed using a split Bregman method for total variation denoising via the scikit-image library.^33^^,^^34^ The denoising method regularization parameter was set at 0.1 (λ=0.1).

OCT images were first processed for segmentation and VF layer recognition. Images consisted of a heterogeneous configuration of grayscale pixel intensities based on the structural properties of the epithelium (EP) and lamina propria (LP). This pixel intensity distribution was utilized to establish thresholding values within each frame to differentiate image noise and non-tissue pixels (e.g., air) from different VF layers. Image intensity histograms were constructed for each frame which generally contained three intensity peaks: signal noise, EP, and LP. Based upon the overlay of hundreds of pixel intensity histograms, an algorithm was designed and implemented for dynamic intensity-based thresholding. Histograms were individually smoothed using quadratic univariate splines from the SciPy library to maintain characteristic peaks of the original plots. Peaks were separated by identifying local minima (troughs) in the splines. Values identified for separating peak regions in histograms were utilized as thresholds to differentiate VF layers and noise. In the case of a suboptimal quality OCT frame (e.g., low signal-to-noise ratio) which did not display characteristic peaks, default separation ratios predicted a thresholding value for the frame. EP and LP automated contour recognition was achieved using scikit-image’s function find_contours,^35^ an implementation of the marching squares algorithm.^36^ The basement membrane (BM) was inferable as the region between the EP and LP contours. Irrelevant contours, produced by noise left after filtering or partial structures not of interest, were removed computationally to first isolate the presumed LP and then the EP of each VF, and the contours were further stabilized by alignment. A summary of the image processing workflow has been included in Fig. S1 in the Supplementary Material.

### 2.5 Analysis: Vocal Fold Thickness and Vertical Displacement

Following segmentation, the thickness of the EP, LP, and combined EP/LP layers throughout the VF contour was measured. Of note, the OCT signal strength wanes as it nears its maximum tissue penetration depth of ∼1.0  mm and, as a result, differentiation between the superficial, intermediate, and deep LP sublayers and identification of the vocalis muscle was not feasible; additional parameters in software cannot repair the rapid and uniform loss of physical signal for digital differentiation of these layers. In this study, with respect to image analysis, the term LP refers to the detectable LP which likely includes the superficial LP and some elements of intermediate and deep LP. On OCT images, the deep border of the LP was segmented at a definite transition zone between VF tissue and the noise layer.

VF layer depth was calculated from the segmented contours by automatically measuring the pixel count within each A-line along the VF contour and converting it to metric units using the axial resolution (4.24  μm/pixel). Thickness measurements were recorded during the normal respiratory cycle and during phonation. Surface tracings of the EP and LP were plotted through time in 3D to depict VF motion. Finally, the vertical shift (y axis) of the mucosal wave was calculated by selecting the superior-medial most point on each VF and measuring displacement between consecutive frames. Vibration amplitude is determined by calculated displacement from the position of the VF at rest immediately before phonation. Numerically, the resolution is determined by the pixel density of the imaged VFs in tandem with the pixel to μm conversion ratio in the axial and lateral directions, which are described in Sec. 2.8.

### 2.6 Analysis: Depth-Resolved Videokymography

Space-time plots of VF thickness were constructed to depict morphological change within the individual VF layers during phonation. Thickness measurements of the EP, LP, and combined EP/LP layers along the superior surface of the VF were translated to color maps. Data from sequential OCT frames were plotted together to demonstrate VF thickness in spatial position (x axis) and time.

### 2.7 Analysis: Space-Time Vocal Fold Velocity and Vector Mapping

**Fig. 3:**
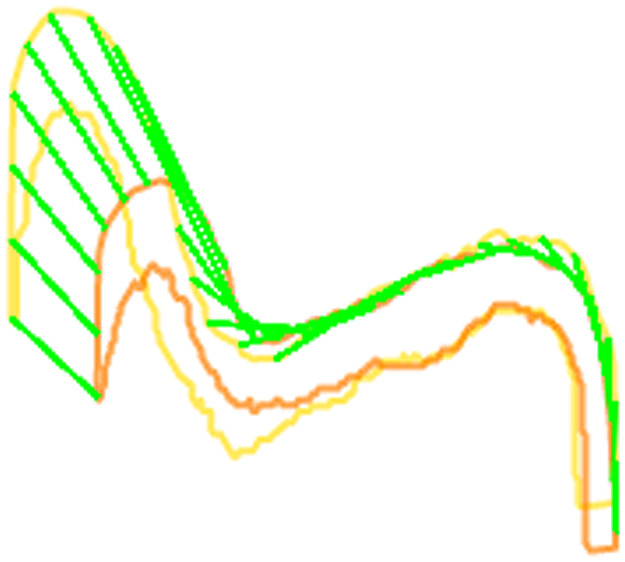
Demonstration of VF displacement tracking. Two EP segmentations from consecutive image frames are shown in yellow and orange. Green lines visually demarcate the displacement vector between the 30 equidistant points along the superior surface of each segmentation. Subsequent calculations for velocity and vector mapping are based on these displacement vectors.

Vibrational velocity along the surface of the VF was calculated. For each frame within a data set, 30 points were distributed equidistantly along the width (x axis) of the superior contour of each VF. The x axis (horizontal wave) and y axis (vertical wave) displacements of each point were measured between consecutive frames (Fig. 3). Using the system frame rate (250 Hz), velocity in the transverse and vertical axes was calculated for each point. The horizontal and vertical velocities were separately color mapped and plotted with respect to time. Additionally, the net, two-dimensional (2D) velocity vector was calculated for each point and plotted with respect to time to depict the magnitude and direction of motion at different regions of the VF.

### 2.8 OCT Measurement Validation

The VF thickness and displacement measurement techniques described in this study were validated for accuracy. The OCT system hand piece was secured upright on a stable, flat surface with the light signal reflected vertically downward. A linear translation stage with 10  μm readable resolution (TSD-3, OptoSigma, Santa Ana, CA, USA) with a protected silver mirror (PF10-03-P01-Ø1″ Protected Silver Mirror, Thorlabs, Newton, NJ, USA) on the stage surface was positioned directly underneath the signal. The stage height was positioned to align the image of the mirror at the top of the OCT frame. Next, OCT images of the mirror were sequentially captured as the stage was translated inferiorly in 0.5 mm increments away from the tip of the GRIN rod. Twenty-five incremented images were captured within the OCT vertical frame as the stage was translated inferiorly a total of 12 mm.

Accounting for the approximate refractive index in tissue (1.4), the approximate axial and lateral resolutions of our OCT system were calculated to be 4.24 and 10.625  μm/pixel, respectively. Using ImageJ, the pixel count (y axis) from the top of the OCT frame to the surface of the mirror, represented by the superior-most horizontal line of hyperintense pixels, was measured. The axial resolution was used to calculate distance between the GRIN rod and the mirror. Measured distances were compared with metric distance values from the linear stage. A linear regression model based on metric distance (independent variable) and OCT pixels (dependent variable) demonstrated a correlation of $R^{2}>0.999$ (p<0.05). Statistical analysis was performed using SYSTAT v13.0 (San Jose, CA). Validation was not done with cadaveric larynges due to the known artifacts associated with conventional histological techniques,^37^ which would lead to inaccurate measurement validations.

## 3 Results

VCSEL OCT was performed on 33 patients (ages 28 to 62; Video S1). Twenty participants were female (61%) and 13 participants were male. Twenty-one participants with high-quality cross-sectional OCT images of the VF were included in the analysis. Twelve participants’ data sets were excluded because of low signal-to-noise ratio, suboptimal substructural resolution, incomplete width of VF within the OCT frame and/or insufficient consecutive OCT frames during phonation. There were no complications associated with imaging.

### 3.1 Interpretation of OCT Images

As light propagates through biological tissue, some photons are absorbed while others are backscattered. The intensity of backscattered signal depends upon optical properties (e.g., absorption, scattering, anisotropy, and refractive index) of tissue components along the signal pathway in both forward and backward directions. As OCT is interferometry-based, the substructural composition of different tissues dictates the grayscale pixel intensity in OCT images. Dense, highly scattering tissues (e.g., collagen-rich muscle) reflect more signal and have higher pixel intensity (near-white or white) while tissues with looser consistencies appear darker.

### 3.2 Image Analysis

**Fig. 4:**
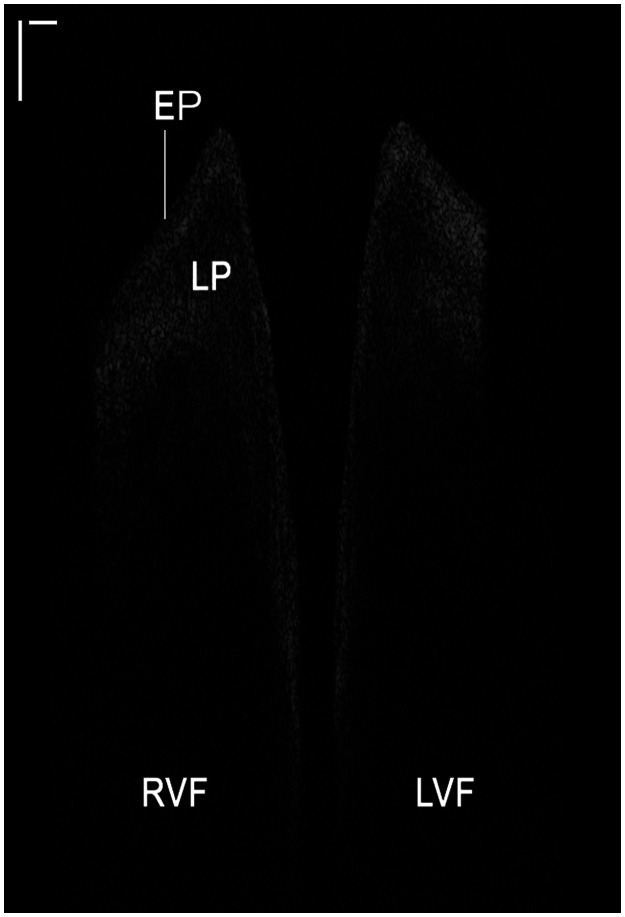
OCT coronal plane, single frame. Cross-sectional OCT image of the VF in the coronal plane, with delineation of superficial VF layers. Scale bars denote 500  μm axially and laterally; scaling was adjusted for visual presentation. RVF, right vocal fold; LVF, left vocal fold; EP, epithelium; and LP, lamina propria (Video S2, 36.18 MB, MP4 [URL: https://doi.org/10.1117/1.JBO.26.8.086005.2]).

**Fig. 5:**
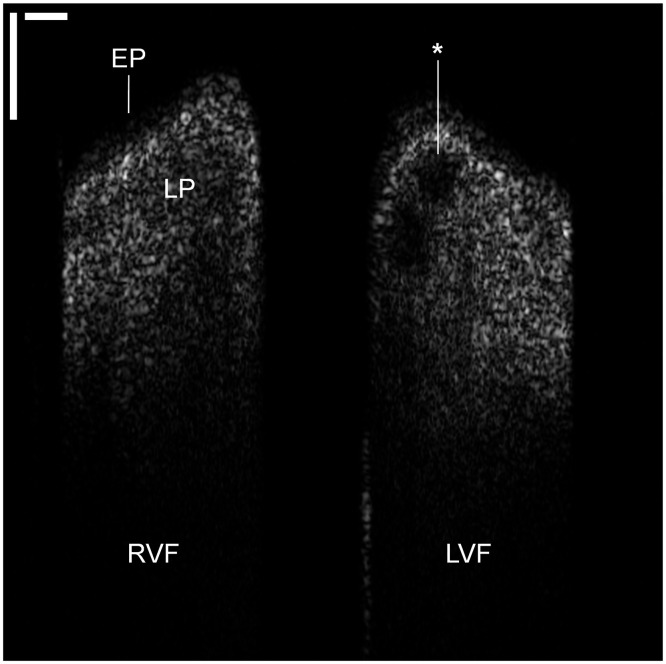
OCT coronal plane with subepithelial lesion, single frame. Cross-sectional OCT image of the VF depicting a subepithelial hypodensity (asterix) in the LVF. Scale bars denote 500  μm axially and laterally; scaling was adjusted for visual presentation. RVF, right vocal fold; EP, epithelium; and LP, lamina propria.

Thirty-nine data sets from 21 participants featured high signal-to-noise ratio and optical penetration depth to permit image analysis. The layered VF architecture including EP and LP were identified. Figure 4 shows a single OCT frame (modified 2.50:1 width-to-height aspect ratio) depicting the VF in the coronal cross-sectional plane; substructural VF layers are labeled according to their respective, variable optical properties. The increased collagen density and microvasculature in the LP results in increased optical scattering and higher signal intensity in comparison to the EP. As maximum OCT signal penetration depth is ∼1.0  mm in tissue, the back scattered light drops beyond the superficial LP into the deeper, more loosely-organized intermediate LP and beyond. For one particular subject, all OCT frames consistently depicted a subepithelial, round hypodensity in the LP of the LVF (Fig. 5) in all frames of the data set. However, no surface pathology or contour irregularities were noted on endoscopic video. From image analysis, it was determined that while the average EP thickness of the LVF and RVF were comparable with a difference of −4  μm, average detectable LP thickness differed by −116  μm and vertical displacement by −128  μm. The image findings may represent early stages of a VF cyst, and in tandem with the analytical findings, they allude to the potential for OCT-based identification and surveillance of benign and malignant VF lesions in a non-invasive manner.

Video S2 depicts consecutive OCT frames at the system’s native imaging speed (250 Hz) and at delayed speed (12 Hz) to demonstrate cross-sectional VF vibration in the coronal plane. The fundamental frequency of the human VF ranges from 90 to 180 Hz in males and 160 to 260 Hz in females. Our VCSEL OCT system maximum frame rate (250 Hz) is below the Nyquist frequencies of most males and females. Hence, aliasing occurs and the LVF and RVF are depicted out of phase in some OCT frames. Nonetheless, with consecutive phonation over a period of at least 3 s, the vertical mucosal wave can be visualized and measured.

### 3.3 Vocal Fold Thickness and Vertical Displacement

The EP and LP thickness was calculated during the normal respiratory cycle and during phonation. LVF and RVF layer thicknesses are detailed in Table 1, with subcategorization by gender. The data distribution was further analyzed for skewness by excluding data points beyond three standard deviations (SD) above and below the mean, as highlighted in Table 1. On image analysis, it was noted that the EP and LP layers underwent mechanical stretching at the medial most point of the VF during phonation. Hence, these regions yielded higher thickness measurements compared to the lateral and inferior VF surfaces.

**Table 1:** OCT-based VF thickness and vertical displacement measurements during the normal respiratory cycle (resting state) and phonation. Results are reported for all points along the VF surface (“full distribution”) and an outlier-excluded distribution within ±3 SD of the sample mean (“select distribution”). Vertical displacement in the resting state is representative of VF which is not actively phonating. However, resting VF changes in surface curvature depending on their position at any given point in time, including adducted, abducted, tensed, paramedian, and fully open. In addition, there is residual motion—bulk, artifact, or otherwise—that remains unresolved. EP, epithelium and LP, lamina propria.

		VF thickness, full distribution (μm; mean ± SD)		VF thickness, select distribution (μm; mean ± SD)		Vertical displacement (mm)
		EP	LP	EP	LP	
Resting state	Total (n=11)	102 ± 59	414 ± 215	129 ± 61	409 ± 208	0.00044
	Male (8)	111 ± 61	367 ± 197	106 ± 49	367 ± 197	0.00049
	Female (3)	67 ± 24	598 ± 182	66 ± 24	595 ± 179	0.00020
Phonation	Total (n=13)	85 ± 55	456 ± 202	80 ± 36	445 ± 183	1.33
	Male (6)	83 ± 42	379 ± 136	81 ± 35	376 ± 130	1.38
	Female (7)	87 ± 66	525 ± 225	79 ± 38	522 ± 220	1.24

In the coronal plane, the superior-medial most point on the VF contour was identified and used to measure the vertical (y axis) displacement of the VF within the mucosal wave. During phonation of the letter “e” in a normal speaking volume, the mean displacement in the vertical axis was 1.33 mm for all participants, 1.38 mm for male participants, and 1.24 mm for female participants (Table 1).

### 3.4 Depth-Resolved Videokymography

**Fig. 6:**
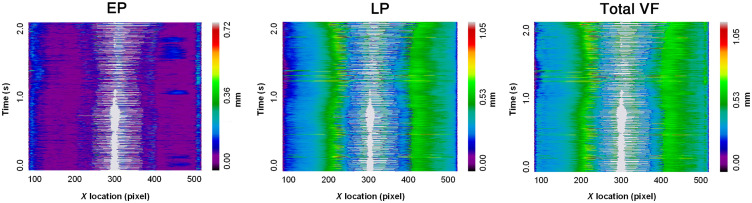
Depth-resolved 2.5-s VKG plot. Participant A: depth-resolved VKG plot of the VF EP, LP, and combined EP/LP layer heights per A-line throughout a sustained phonation of 2.5 s.

**Fig. 7:**
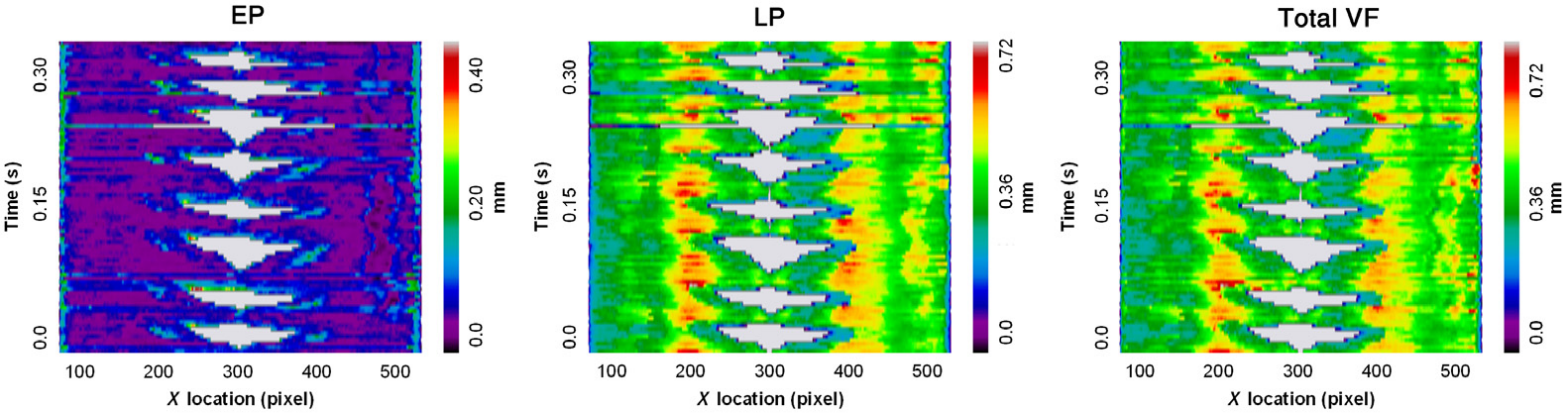
Depth-resolved 0.4-s VKG plot. Participant B: depth-resolved VKG plots of the VF EP, LP, and combined EP/LP layer heights per A-line throughout a sustained phonation of 0.4 s.

Color maps of EP, LP, and combined EP/LP thicknesses in consecutive OCT frames were organized into space-time plots. Depth-resolved VKG plots visually represent the glottal waveform in a similar manner to traditional VKG with the added element of subepithelial, substructural VF morphology. This permits the clinician to assess subepithelial changes at full VF abducted and adducted states and compare substructural properties between the LVF and RVF. Figure 6 depicts representative depth-resolved VKG plots from a single subject for 2.5 s of consecutive phonation. In these maps, which analyze tissue as seen by A-lines, the color spectrum represents VF layer height, and light gray corresponds to air and represents the glottal opening or change in the free margin of the VF over time. A similar kymogram from a second subject was cropped to illustrate phonation for 0.4 s (Fig. 7), providing a more close-up view of the alternating layer heights during individual wave cycles as captured by the OCT system. In both Figs. 6 and 7, the approximate periodicity of the VF mucosal wave is appreciated, allowing for objective and visual assessment of mucosal wave symmetry across the larynx.

### 3.5 Space-Time Vocal Fold Velocity and Vector Mapping

**Fig. 8:**
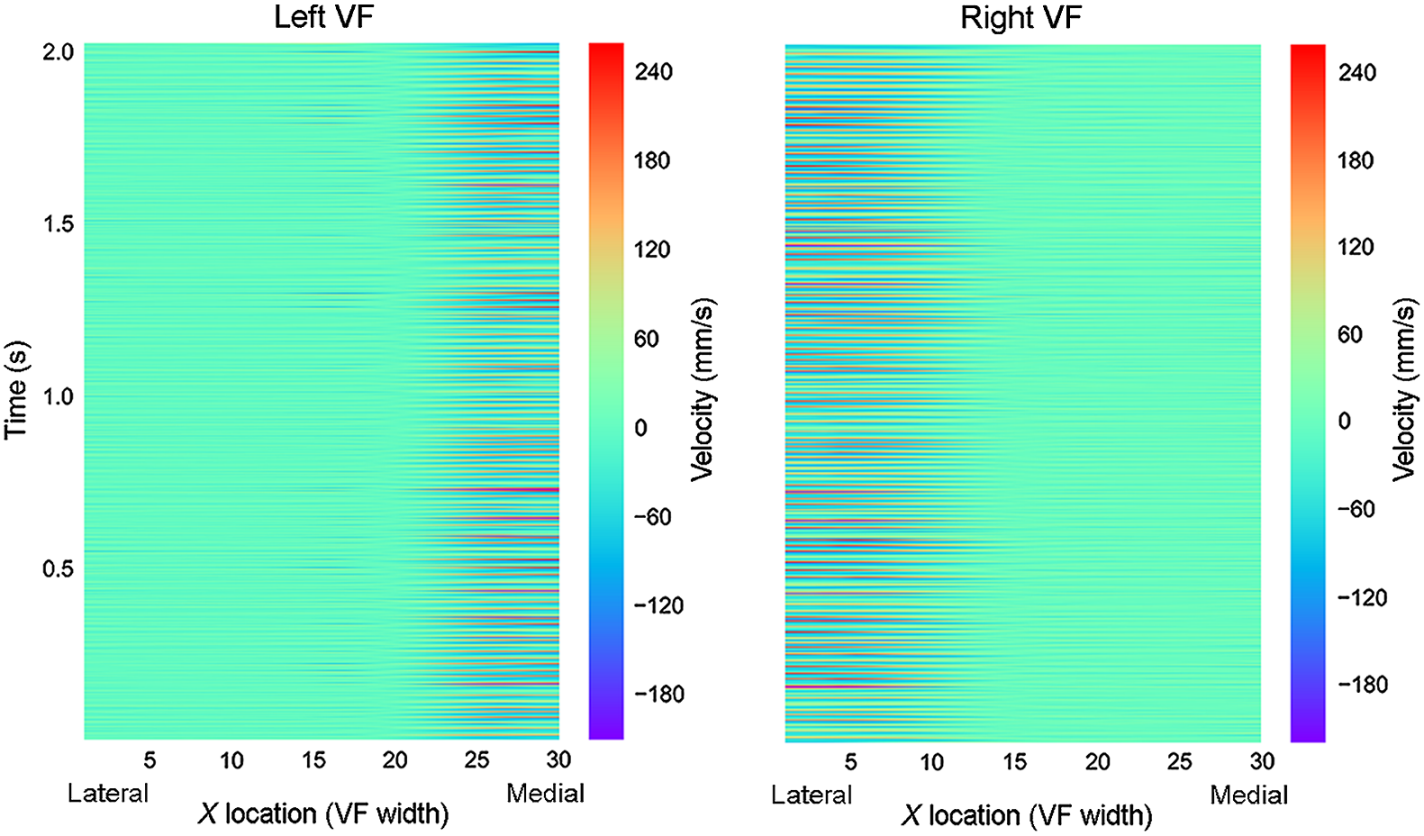
Space-time resolved velocity plot. Participant A: space-time resolved VF velocity along the surface of the VF during a sustained phonation of 2.5 s.

**Fig. 9:**
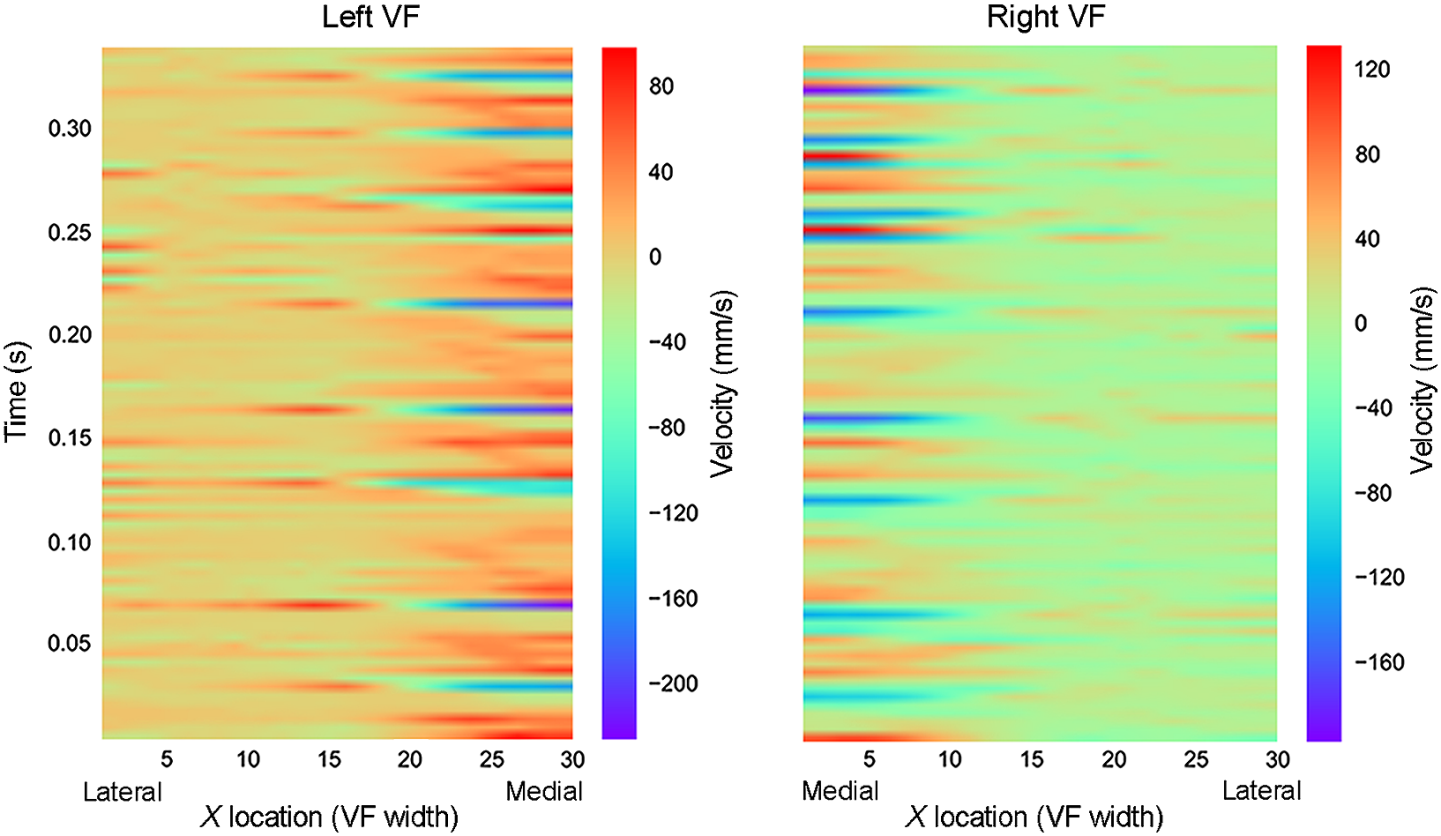
Space-time resolved velocity plot. Participant B: space-time resolved VF velocity along the surface of the VF during a sustained phonation of 0.4 s.

Velocity vectors at 30 equidistant points along the surface of each VF fold were calculated for each OCT frame in a sequence of phonation. Analogous to the bellows of an accordion, adjacent points along the VF surface move in relation to one another as anatomically distinct regions of the VF stretch in different vectors. The horizontal (x axis) and vertical (y axis) velocities were calculated and represented separately in color maps. Figures 8 and 9 depict space-time resolved vertical velocity plots for two separate patients. In these plots, color intensity represents velocity. The x axis of each plot indicates the relative position along the surface of the VF, with points 30 and 0 representing the medial edges of the LVF and RVF, respectively. As depicted in Figs. 8 and 9, the medial, free edges of the VF vibrated at the highest speeds. We also noted that VF surfaces extending as far lateral as the glottis-supraglottis junction did feature minute y axis displacement, highlighting the spatial range and magnitude of the vertical component of the mucosal wave. For each data set, the horizontal velocities at each VF point were also separately color mapped but not presented here. These figures permit focused analysis of vibrational patterns and irregularities with the horizontal and vertical mucosal waves within the image plane.

**Fig. 10:**
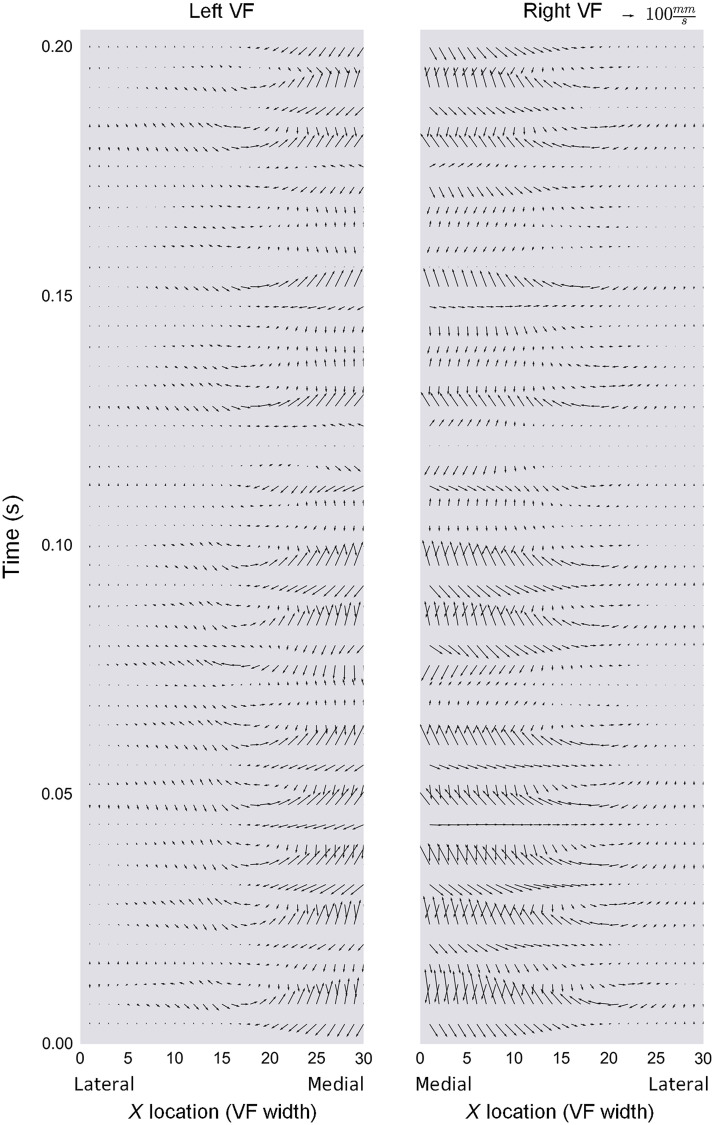
Vector map during phonation. Participant A: spatially resolved net 2D vibrational speed (mm/s; magnitude of arrow) and direction during phonation. All directions are shown from the coronal plane: arrows toward the center of the image represent velocities in the anatomic medial direction, whereas those toward the outer edges left and right denote respective anatomic lateral directions. Arrows toward the top of the image indicate anatomic superior direction, and those toward the bottom of the image indicate anatomic inferior direction. A smaller segment of time compared to that of Fig. 8 has been visualized for increased legibility of vector arrows.

**Fig. 11:**
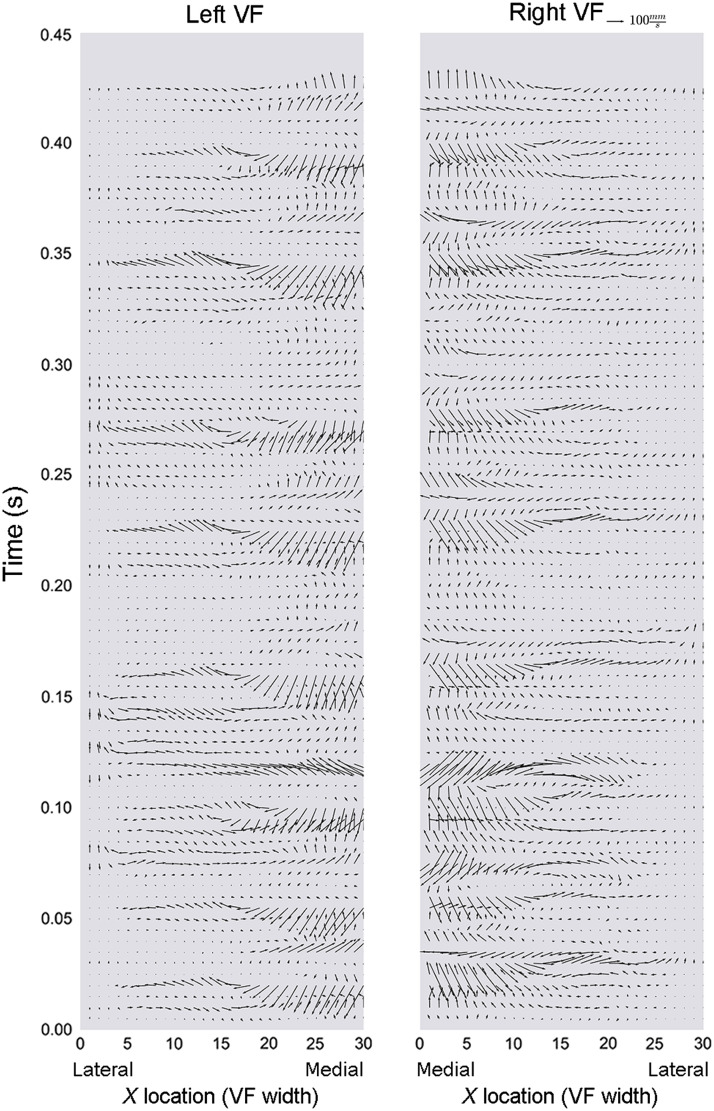
Vector map during phonation. Participant B: spatially resolved net 2D vibrational speed (mm/s; magnitude of arrow) and direction during phonation. Arrow directions are depicted in the same manner as Fig. 10.

For each of the 30 points along the VF surface, the magnitude and direction of the combined (horizontal and vertical) 2D velocity vectors were plotted with respect to time. Figures 10 and 11 are representative vector plots for a 0.5-s timeframe for the same two patients represented in Figs. 8 and 9, respectively. The magnitude of the arrows represents net 2D vibrational speed (mm/sec) and the direction of the arrows represents the velocity vector. Vector maps allowed for visual representation of how spatially relative points along the VF surface behave during the mucosal wave.

## 4 Discussion

True depth-resolved analysis in tandem with surface kinematic characterization of phonating human VF *in vivo* has not been done before; the technology to accomplish this simply was not available previously. Contemporary functional imaging of the vibrating VF is limited to the superficial, superior surface and relies on the available modalities of videostroboscopy, HSDI, and VKG. Each of these has limitations. Videostroboscopy is the workhorse for imaging the mucosal wave, but it does not detect aperiodic vibrations. HSDI can capture superficial mucosal waves and is not limited by aperiodicity, but it is associated with an enormous data burden and delayed image processing. Meanwhile, VKG is fundamentally limited to linear imaging along a transverse axis of the glottis, though adaptations of VKG including multi-line kymography^38^ and 2D scanning VKG^39^ have been described to expand VKG imaging along the anteroposterior dimension of the VF. Most importantly, all of these modalities are limited to visualization of the superior VF surface in the standard laryngoscopic view, compressing 3D surface motion data into 2D images without depth resolution.^11^

Multiple groups have described laser-based stereotriangulation and surface mapping to measure vertical displacement along the superior VF surface during vibration *in vivo* in human participants^40^ and *ex vivo* in human cadaveric larynges.^41^ While innovative in capturing vertical displacement of the mucosal wave along the superior VF surface, transoral laser stereotriangulation—and surface mapping—do not permit depth-resolved imaging of the subepithelial layers and inferior VF surfaces which constitute the true vertical mucosal wave.^40^^,^^42^

Recently, Chettri’s group described *in vivo* 3D geometric mapping of the medial VF surface in canine larynges to evaluate VF contour changes following selective activation of intrinsic laryngeal muscles.^43^^,^^44^ However, these induced morphometric changes were not representative of the native mucosal wave and the 3D VF reconstructions did not depict the superior VF contour in coronal section. Moreover, the described technique involved near-contact imaging after hemilaryngectomy and would not be feasible for *in vivo* imaging of the medial VF surface in normal humans. Hence, established and most investigational laryngeal imaging tools remain limited to VF surface imaging with incomplete characterization of both the horizontal and vertical mucosal waves.

### 4.1 Computational Algorithm-Driven New Understandings of the Mucosal Wave

In 1975, Matsushita first coined the term “mucosal wave” by likening the vibratory undulation of the VF to waves in a fluid medium.^45^ Nearly a half-century later, our understanding of mucosal wave biomechanics remains largely based on *ex vivo* animal or human cadaveric laryngeal studies,^41^ *in vivo* high-speed imaging of VF surface,^40^ or laryngeal models.^9^ As such, *in vivo* vibration of the subepithelial VF layers, which are fundamental in mucosal wave production and pitch modulation, has not previously been quantified in human subjects.

In this report, we present objective OCT-based automated algorithms and resulting depth-resolved analytics alongside surface kinematics of the human mucosal waveform in the vertical plane, thereby giving insight into depth-resolved biomechanical properties of VF layers during phonation. OCT provides direct imaging of the vertical mucosal wave in coronal section, which can be more accurately quantified by measuring the amplitude (y axis shift) of the medial, free edge of the VF. We estimated a mean vertical amplitude of 1.33 mm at the medial edges of both VF. Sommer et al.^46^ described HSDI coupled with stereo-matching algorithms to estimate inferior-superior VF kinematics, however, these were limited estimates based on computer stereo vision. In comparison, George et al. reported mean vertical amplitudes of 0.7 mm (LVF) and 1.2 mm (RVF) using laser triangulation, with an inexplicable disparity in measurements across the glottis.^40^

There are differences in vertical amplitude values between the values reported here and those of previous studies, some of which may be attributable to biological variation. Imaging as reported in this study and those of previous reports have all challenged by bulk motion and other sources of motion artifact. However, in this study, stabilization was largely achieved as evidenced by the minimal vertical motion detected for VF in the resting state (Table 1). Previous methods relied on algorithms to indirectly estimate vertical displacement from stabilized 2D transverse plane images. In contrast, OCT depth-resolved images observe motion from the coronal plane, which thus directly captures vertical displacement of the VF and provides a more accurate assessment of vertical motion.

*In vivo* laryngeal ultrasound has also been described to estimate lateral and vertical VF amplitude during phonation in human participants. Tang et al.^47^ combined ultrasound and EGG to estimate the vertical amplitude of the underlying body layer between 0.32 and 0.51 mm. Although lateral vibrational amplitude was also reported (0.059 to 0.09 mm), they acknowledge that lateral vibrational amplitude is very minute and “can hardly be directly extracted from 2D ultrasonic images because of the limitation of image resolution.” Furthermore, transcutaneous ultrasound is limited by relatively lower signal-to-noise ratio, as intervening soft tissue and, importantly, the acoustically dense thyroid cartilage modulate the sound wave.

Both the horizontal and vertical velocities of the mucosal wave surface were evaluated by measuring x and y axes amplitude at multiple points along the VF contour, respectively. More importantly, these tracked velocities can be correlated to underlying changes in geometry. The EP and LP thickness reported in this study represent the first *in vivo* detailed thickness measurement of distinct human VF layers during natural phonation. Hu et al.^48^ used high-frequency ultrasound to measure thickness of the entire true VF (4.25 to 6.22 mm) in awake human participants, however, delineation of individual VF layers was not feasible given ultrasound spatial resolution limits. Our EP (129±61  μm) and LP (409±208  μm) measurements were comparable with previously published OCT measurements of static VF and histology-based measurements of 80 to 100  μm for EP and 600 to 700  μm for SLP, albeit these OCT studies were performed at rest or under sedation.^25^^,^^49^^–^^52^ It should be noted that the true thickness of the entire LP layer, down to the LP-vocalis muscle interface, cannot yet be ascertained by our current system design due to OCT hardware limitations with depth of signal penetration. From the perspective of image analysis alone, however, the consistent LP segmentation results obtained in this study suggest that once OCT systems are capable of deeper signal penetration levels, the software should be able to effectively segment the images produced. It should also be noted that the mean and SD of LP measurements (409±208  μm) are affected by biological variation between participants and differences in LP morphology depending on the relative location along the transverse axis of the glottis, with thinner LP at the medial VF and thicker LP laterally. While recognizing the limiting effects of signal loss, we are yielding the first morphometric estimates from large quantities of measurements for the LP layer of the VF.

### 4.2 Purpose and Necessity of OCT for Data Acquisition

OCT is widely utilized in ophthalmologic^53^ and cardiovascular^54^ imaging and is now rapidly traversing the technological curve for application in laryngology. Innovation in laryngeal OCT technology over the last two decades has led to progressive improvements in imaging speed, resolution, and range. Although *in vivo* transoral cross-sectional imaging of the vibrating VF has been previously demonstrated, these early OCT systems were limited by low resolution and imaging speeds, precluding any meaningful clinical utility.^8^^,^^15^^,^^17^^,^^20^^,^^55^ The use of a VCSEL swept-source laser enhances office-based OCT of the larynx by increasing the imaging range to up to 20 cm while maintaining micrometer-level resolution.^56^ In 2016, we first reported office-based, transoral VCSEL OCT with panoramic, high-resolution images of phonating VF in coronal cross-sectional plane.^25^ In that study, optical Doppler tomography (ODT) was described to estimate vibrational velocity by calculating the Doppler shift between adjacent A-lines on consecutive OCT frames.^25^ ODT, however, has a limited quantifiable velocity range due to phase wrapping and phase washout and computes vibrational velocity in the y axis only as the VF oscillates toward and away from the light source.^57^ Furthermore, ODT presents a significant computational burden, only to yield phase differences in motion propagation at a single point along the VF. Further research efforts to achieve *in vivo* cross-sectional imaging of the VF have been described with conventional B-mode and ultrafast plane wave ultrasonography, which are capable of imaging speeds up to 800 and 10,000 Hz, respectively.^48^^,^^58^ Ultrasound, however, features significantly limited lateral resolution and hence cannot reliably image distinct VF layers.^58^ Thus OCT remains the only *in vivo* imaging tool to delineate subepithelial VF layers with micrometer resolution during phonation.

### 4.3 Necessity of Computational Algorithms as Introduced

The development of VCSEL OCT technology and accompanying automated analytical methods, as introduced here, offers a necessary baseline for methods to close the knowledge gap for mucosal waves. Challenges in image data processing do not only include the sheer quantity of images to segment and evaluate. To the human eye, the contrast difference between EP and LP layers appears to be low in OCT images. This may raise questions regarding the repeatability and reliability of automatic segmentation. However, it is these subtle differences in contrast which confer a necessity for automatic segmentation method over manual segmentation. False color is often used to make images more comprehensible to the human eye, but this type of rendering introduces bias in expectations and viewable results. Standardized algorithms, by comparison, can objectively identify subtle but substantial differences in contrast beyond the capabilities of the human eye,^59^ which can also have its perception unwittingly affected by phenomena such as White’s illusion.^60^ Furthermore, studies using VCSEL and other OCT data have quantitatively observed unique pixel intensity profiles for various VF tissue structures.^6^^,^^26^

Our results demonstrate that automatic segmentation was able to distinguish a consistent pattern of multi-modal distributions of pixel intensities across OCT images. In our study, all measurements were calculated using a pixel-to-micron scale determined through calibration measurements in air with an accommodating refractive index factor for tissue. Although we could not justify collecting VF tissue specimens from healthy volunteers for confirmation of our measurements in this study, numerous studies using human and animal models have extensively demonstrated the validity of VF tissue measurements collected using OCT in direct comparison to that of the existing gold standard: histology.^7^^,^^37^^,^^49^^,^^61^

The contouring algorithm itself had been validated through visual inspections of segmentation results for hundreds of images from various data sets by otolaryngologist head and neck-surgeons with experience in OCT image acquisition and analysis. Moreover, the automatic segmentations and subsequent measurements in this study were localized to the correct region of the image relative to the rest of the anatomy and were in agreement with previously reported OCT measurements of human VF.

### 4.4 Practical Applications of this Technology

Since the seminal work of M. Hirano, efforts to visualize and analyze the mucosal wave *in vivo* have been largely restricted to indirect imaging of the VF surface from the standard laryngoscopic perspective (superior surface) of the VF. Considering the 3D form of the VF and the horizontal and vertical components of the mucosal wave that propagate with airflow through the larynx, different regions of the VF vibrate with different amplitudes which can, in turn, be modulated during the phonation process. Spatially resolved velocity measurement and kymographic representation, as presented here, offer unique, objective data on the mucosal wave to enable greater understanding of how different VF regions and substructural layers behave during the mucosal wave. This insight would, may in turn, aid in understanding the impact of benign or malignant VF lesions on mucosal wave mechanics. Betz’s and Aren’s groups (Germany) have independently and jointly published formative studies on *in vivo* OCT imaging of benign, premalignant, and malignant and laryngeal lesions with diagnostic confirmation by biopsy and histopathological analysis.^62^^–^^64^ OCT was shown to have a high predictive accuracy (79% to 93%) for histologically confirmed carcinoma based on identification of basement membrane disruption. Although dysplastic lesions directly abutting the basement membrane may be more challenging to differentiate from carcinomas, Kraft et al.^64^ reported the exact grade of dysplasia could be accurately predicted in up to 71% of precancerous lesions. These *in vivo* studies were performed on static VF and suggest OCT’s potential as a valuable adjunct diagnostic tool for evaluating VF lesions.

Given the mucosal wave involvement of both superficial and deep VF layers, it can be inferred that benign and malignant VF lesions, with varying depths of VF penetration, would affect mucosal wave mechanics in different ways. A tension-producing VF lesion (e.g., fibrosis) may abnormally increase the fundamental frequency, while a load-producing VF lesion (e.g., Reinke’s edema) may result in a pathologic decrease in fundamental frequency.^65^ As cancers are known to be firmer (desmoplasia) than native tissue, tissue mechanics can thus be used as a means of contrast for tumors, as vibratory behavior locally would change and be detectable through automated means. Hence, methods for quantitatively analyzing cross-sectional OCT imaging of VF vibrational parameters, as introduced here, can potentially expand our understanding of the functional consequences of VF lesions and represent an effective and efficient means of differentiating benign and malignant pathologies.

### 4.5 Study Limitations and Future Directions

A limiting factor of our study is the sample population. Herein, ∼50% of participants were screened out due to unfavorable oropharyngeal anatomy and intolerance of trans-oral endoscopic imaging (e.g., gag reflex). Participant movement and hand tremor of the operator can also contribute to image noise and artifact. To optimize our images, motion artifact was corrected for using the template matching and stabilization methods described in our methodology. As commercial optical hardware continues to improve in performance while shrinking in size, future developments of our system will include improving the form factor to increase patient tolerance for trans-oral imaging, which will improve the sample size.

Our clinical imaging technique (Video S1) results in a degree of motion artifact, as the imaging device is held by the operator while the participant self-stabilizes in an upright seated position. Furthermore, as our hardware permits imaging across a single-line transverse to the VF axis, we are not able to image the entire VF region. Hence, with motion orthogonal to the scanning direction, the VF surface points in a given frame cannot be spatially correlated among all frames in an image sequence with complete accuracy. The same critique can be applied to existing technology such as depth VKG. In the short term, more robust stabilization could involve image alignment algorithms that reference the conventional endoscope images captured simultaneously by the handpiece. With future advancements in OCT systems and methodology, the ability to image the entire VF structure and consequently align consecutive frames would allow us to better correlate surface points within an image sequence and further expand our data on VF kinematics. The potential is there: volumetric imaging with full-field OCT has been completed with human skin *in vivo*^66^ and laryngeal specimens from pediatric cadavers.^67^

A notable technical limitation of our OCT system is the sampling rate of 250 Hz which is below the estimated Nyquist frequency of females (∼500  Hz) and most males (∼250  Hz). This results in aliasing and influences the representation of the glottic vibratory cycle on 3D VKG plots. Previous studies have described concurrent HSDI with EGG to compute glottal geometry in *ex vivo* canine and human larynges.^68^^,^^69^ Hence, a solution to extrapolate the mucosal waveform would be to computationally synchronize or gate EGG measurements with OCT data. The sub-Nyquist sampling also explains why the LVF and RVF are often depicted out of phase on individual OCT frames (Video S2). In a series of OCT images captured during sustained phonation, individual, albeit non-consecutive, frames from the wave peaks and troughs can be identified to recreate the full periodic cycle and measure the net vertical VF displacement during vibration. Although our VCSEL OCT system is theoretically capable of imaging at speeds >250  Hz, we discovered this would occasionally overclock the galvanometer scanning motor and cause the system to malfunction. In the future, as sweep rates of VCSEL lasers increase into the megahertz ranges, OCT frame rates will eventually exceed the Nyquist frequency for the fundamental frequency and harmonics of the human voice, permitting imaging of each complete periodic waveform.

With improved imaging data, we can advance our segmentation and analytical methods in tandem. Beyond signal intensity, additional parameters such as the relative location of individual pixels in each frame, the consistency of intensity and positioning per pixel periodically, and more may be integrated for improved segmentation. Going further into the progressive development of objective and scalable analytical methods, principles of machine learning, especially in computer vision, may be integrated for tissue layer feature extraction and layer delineation as well as characterization and localization of normal versus abnormal kinematics through motion analysis and pattern recognition.

Finally, a fundamental limitation with all OCT imaging application remains the maximum signal penetration depth of ∼1.0  mm, which prevents consistent and high-resolution imaging of all LP sublayers and the underlying vocalis muscle. Hence, the screening and diagnostic utility of this technology is more limited with lesions that extend to the vocal ligament (intermediate and deep LP) or body of the VF. Nevertheless, the proposed computer-aided measurements and assessments would facilitate clinical diagnosis and management of voice disorders as well as benign and malignant laryngeal mucosa pathology.

## 5 Conclusions

As a high-speed, cross-sectional laryngeal imaging tool, VCSEL OCT facilitates our ability to image the mucosal wave *in vivo* and may enhance our current understanding of voice production and sound modulation. The objective analysis and graphical representations of VF vibration introduced in this report represent the first foray into quantifying the mucosal wave in its vertical, coronal-plane representation as well as subepithelial within the VF during phonation. VCSEL OCT has clinical applicability to enhance diagnosis and management of a wide host of functional and neurological voice disorders which affect the mucosal wave. As OCT technology continues to evolve and is more readily adopted as a minimally invasive, point-of-care laryngeal imaging tool, this technology may become highly useful for surgeons, speech pathologists, and voice therapists in the management of voice disorders and structural VF pathologies.

## Code, Data, and Materials Availability

Software code and data used in the research to obtain the results reported in the article can be provided upon request to the corresponding author. No materials were used for the analysis.
