# Supplementary material for: Surface kinematic and depth-resolved analysis of human vocal folds in vivo during phonation using optical coherence tomography
Source: J Biomed Opt. 2021 Aug 19;26(8):086005. doi: 10.1117/1.JBO.26.8.086005 (PMC8374544; doi:10.1117/1.JBO.26.8.086005)
Supplement: Supplementary file 3 [file JBO_026_086005_SD003.pdf]

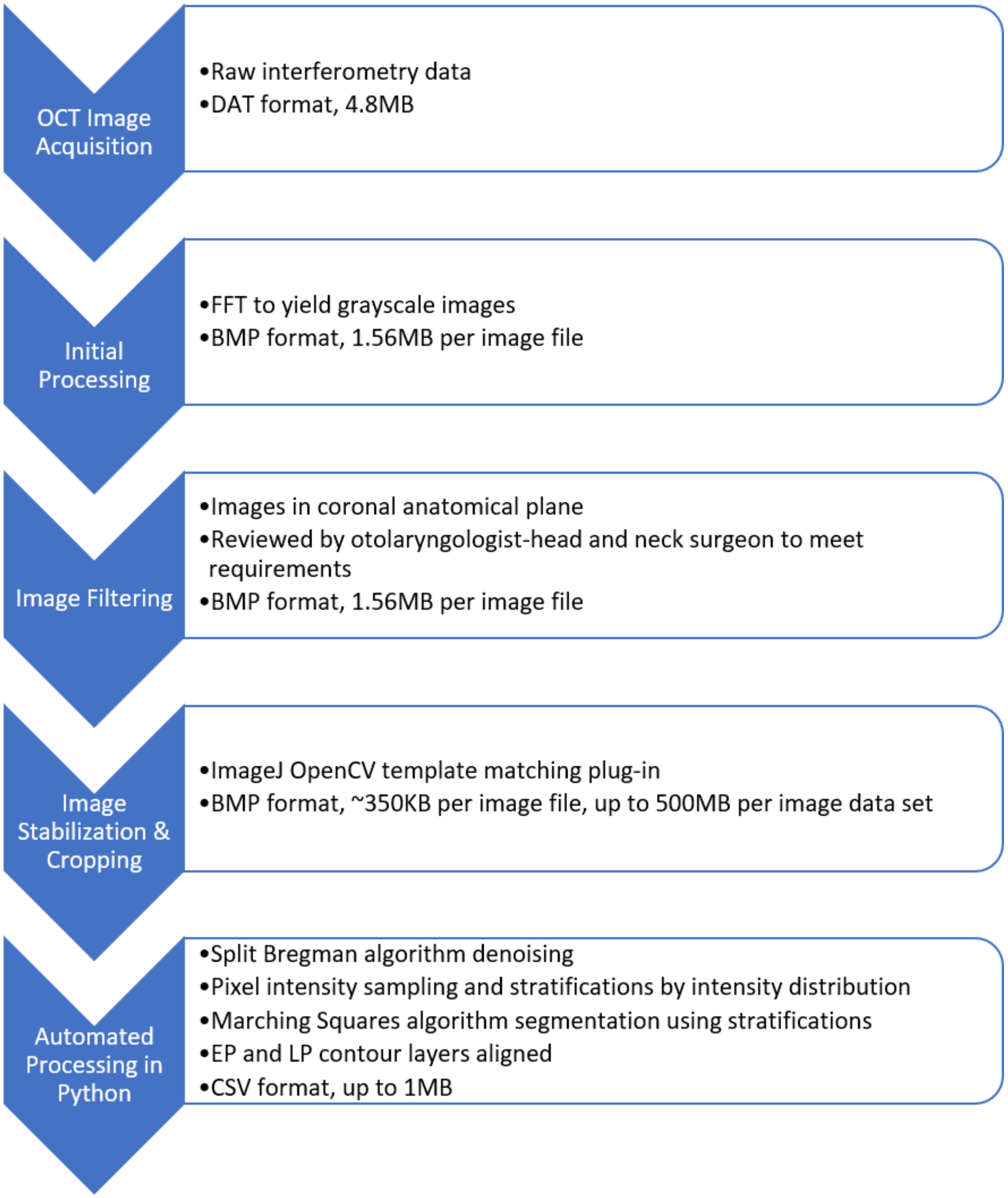

Figure S1. Flowchart representative of initial image processing steps. Major steps and the respective resulting data set and format are indicated.
